# Supplementary material for: The Starch Physicochemical Properties between Superior and Inferior Grains of Japonica Rice under Panicle Nitrogen Fertilizer Determine the Difference in Eating Quality
Source: Foods. 2022 Aug 17;11(16):2489. doi: 10.3390/foods11162489 (PMC9407410; doi:10.3390/foods11162489)
Supplement: Supplementary file 1 [file foods-11-02489-s001.zip › foods-1814295-supplementary.pdf]

Supplementary materials

Table S1 Variance analysis of nitrogen (N) ,variety (V) and position (P) effect on the content of starch, protein and crude fat.

Index	Nitrogen	Variety	Position	N×V	N×P	V×P	N×V×P
Degree of freedom	3	1	1	3	3	1	3
Starch content (%)	138.84**	75.59**	56.48**	6.96**	2.62 NS	0.12 NS	2.64 NS
Amylose content (%)	236.19**	10710.93**	447.11**	0.35 NS	0.94 NS	142.84**	1.96 NS
Amylopectin content (%)	117.62**	833.61**	1056.74**	6.79**	15.71**	83.54**	2.26 NS
Amylose/ amylopectin	314.28**	23118.39**	8738.45**	2.57 NS	19.91**	2893.36**	25.00**
Total protein content (%)	7860.92**	99.37**	8526.15**	67.61**	330.57**	377.56**	119.30**
Albumin content (mg g^{-1})	117.32**	465.22**	392.55**	0.87 NS	1.00 NS	23.16**	0.30 NS
Globulin content (mg g^{-1})	163.67**	4.27 NS	254.26**	2.69 NS	5.46**	19.10**	3.81*
Prolamin content (mg g^{-1})	427.72**	55.15**	67.46**	44.80**	7.82**	0.00 NS	1.55 NS
Glutelin content (mg g^{-1})	207.67**	29.10**	444.14**	199.06**	20.48**	1.36 NS	14.56**
Crude fat content (%)	1308.92**	295.71**	8232.16**	3.30 NS	110.79**	1028.08**	10.77**

*F-value significant at the 0.05 probability level. ** F-value significant at the 0.01 probability level. NS means no significant at $P=0.05$.

Table S2 Variance analysis of nitrogen (N), variety (V) and position (P) effect on starch granule size distribution, amylopectin chain length distribution and crystalline structure.

Index	Nitrogen	Variety	Position	N×V	N×P	V×P	N×V×P
Degree of freedom	3	1	1	3	3	1	3
Small starch granules (%)	427.30**	8842.98**	288.91**	148.75**	52.64**	648.45**	106.26**
Middle starch granules (%)	6941.55**	263975.34**	1102.92**	2756.14**	4138.01**	66060.25**	1826.67**
Large starch granules (%)	3271.13**	80293.20**	16.13**	227.67**	1016.50**	19124.31**	507.88**
Volume mean diameter (μm)	1382.15**	74858.91**	904.36**	56.28**	902.38**	18672.03**	536.77**
A chain (%)	348.18**	44.64**	11.38**	20.99**	9.75**	19.51**	6.56*
B1 chain (%)	0.49 NS	48.90**	0.24 NS	0.49 NS	4.62*	0.13 NS	3.48 NS
B2 chain (%)	15.60*	26.22**	14.70**	2.21 NS	1.39 NS	1.33 NS	2.57 NS
B3 chain (%)	97.35**	124.90**	0.00 NS	31.99**	5.53*	6.85*	5.64*
(A+B1)/(B2+B3)	43.48**	6.76 NS	13.75**	8.80*	40.82**	34.89**	66.58**
Amylopectin average DP	273.89**	10.54*	0.53 NS	6.94*	9.51**	9.75*	6.88*
Relative crystallinity (%)	235.74**	802.71**	101.49**	7.87*	3.71 NS	0.05 NS	0.57 NS
Amorphous (%)	478.51**	4961.74**	352.59**	63.65**	6.12**	76.79**	1.44 NS
Single helix (%)	47.88**	7.68*	215.83**	0.42 NS	6.06**	6.94*	4.02*
Double helix (%)	474.32**	4479.71**	147.28**	56.23**	2.68 NS	47.60**	0.30 NS

*F-value significant at the 0.05 probability level. ** F-value significant at the 0.01 probability level. NS means no significant at $P=0.05$.

Table S3 Variance analysis of nitrogen (N), variety (V) and position (P) effect on starch solubility, swelling power and thermal properties.

Index	Nitrogen	Variety	Position	N×V	N×P	V×P	N×V×P
Degree of freedom	3	1	1	3	3	1	3
Solubility (%)	32.69**	480.04**	59.91**	30.14**	0.11 NS	21.65**	0.49 NS
Swelling power (g g^{-1})	277.62**	5906.85**	719.42**	3.10 NS	3.86*	0.20 NS	6.01**
$\triangle H_{gel}$ ($J \cdot g^{-1}$)	3866.07**	33.51**	326.32**	107.58**	17.90**	22.45**	8.31**
T_O (°C)	16.09**	148.00**	939.69**	990.59**	10.72**	13.73**	62.51**
T_P (°C)	11.35**	291.01**	296.60**	44.49**	13.71**	77.05**	7.22**
T_C (°C)	2.09 NS	452.34**	82.81**	36.16**	0.16 NS	27.76**	0.09 NS
$\triangle H_{ret}$ ($J \cdot g^{-1}$)	303.88**	196.72**	96.99**	18.79**	0.57 NS	0.84 NS	0.44 NS
R (%)	995.21**	96.09**	241.77**	15.85**	1.83 NS	0.46 NS	0.24 NS

**F*-value significant at the 0.05 probability level. ** *F*-value significant at the 0.01 probability level. NS means no significant at *P=0.05*. ΔH_{gel}, gelatinization enthalpy; T_O, onset temperature; T_P, peak of gelatinization temperature; T_C, conclusion temperature; ΔH_{ret}, retrogradation enthalpies; R, retrogradation percentage.

Table S4 Variance analysis of nitrogen (N), variety (V) and position (P) effect on starch pasting properties and rice eating quality.

Index	Nitrogen	Variety	Position	N×V	N×P	V×P	N×V×P
Degree of freedom	3	1	1	3	3	1	3
Peak viscosity (cP)	956.84**	2434.57**	8484.59**	102.05**	87.49**	0.37 NS	30.43**
Hot viscosity (cP)	490.30**	22.29**	3791.95**	169.32**	53.96**	35.80**	20.11**
Breakdown (cP)	16.02**	12435.96**	182.20**	1.08 NS	0.53 NS	66.31**	0.30 NS
Final viscosity (cP)	802.89**	3381.59**	5219.24**	75.77**	83.23**	47.53**	11.98**
Setback (cP)	31.33**	37979.77**	443.27**	4.63*	6.80**	112.22**	8.98**
Pasting temperature (°C)	32.56**	515.56**	36.64**	1.19 NS	7.64**	118.94**	0.74 NS
Taste value	222.86**	10127.70**	102.47**	6.51*	17.44**	16.72**	0.57 NS
Hardness (g)	73.74**	0.74 NS	102.31**	0.41 NS	0.99 NS	1.00 NS	1.15 NS
Stickiness (g)	69.68**	25.85**	1.04 NS	1.17 NS	3.25*	172.93**	1.36 NS

**F*-value significant at the 0.05 probability level. ** *F*-value significant at the 0.01 probability level. NS means no significant at *P=0.05*.
